# The role of champions in the implementation of technology in healthcare services: a systematic mixed studies review

**DOI:** 10.1186/s12913-024-10867-7

**Published:** 2024-04-11

**Authors:** Sissel Pettersen, Hilde Eide, Anita Berg

**Affiliations:** 1 Faculty of Nursing and Health Sciences, Nord university, P.O. Box 474, N-7801 Namsos, Norway; 2 Centre for Health and Technology, Faculty of Health Sciences, University of South-Eastern Norway, PO Box 7053, N-3007 Drammen, Norway

**Keywords:** Champions, Technology implementation, Healthcare personnel, Healthcare services, Mixed methods, Organizational characteristics, Technology adoption, Role definitions, Healthcare settings, Systematic review

## Abstract

**Background:**

Champions play a critical role in implementing technology within healthcare services. While prior studies have explored the presence and characteristics of champions, this review delves into the experiences of healthcare personnel holding champion roles, as well as the experiences of healthcare personnel interacting with them. By synthesizing existing knowledge, this review aims to inform decisions regarding the inclusion of champions as a strategy in technology implementation and guide healthcare personnel in these roles.

**Methods:**

A systematic mixed studies review, covering qualitative, quantitative, or mixed designs, was conducted from September 2022 to March 2023. The search spanned Medline, Embase, CINAHL, and Scopus, focusing on studies published from 2012 onwards. The review centered on health personnel serving as champions in technology implementation within healthcare services. Quality assessments utilized the Mixed Methods Appraisal Tool (MMAT).

**Results:**

From 1629 screened studies, 23 were included. The champion role was often examined within the broader context of technology implementation. Limited studies explicitly explored experiences related to the champion role from both champions’ and health personnel’s perspectives. Champions emerged as promoters of technology, supporting its adoption. Success factors included anchoring and selection processes, champions’ expertise, and effective role performance.

**Discussion:**

The specific tasks and responsibilities assigned to champions differed across reviewed studies, highlighting that the role of champion is a broad one, dependent on the technology being implemented and the site implementing it. Findings indicated a correlation between champion experiences and organizational characteristics. The role’s firm anchoring within the organization is crucial. Limited evidence suggests that volunteering, hiring newly graduated health personnel, and having multiple champions can facilitate technology implementation. Existing studies predominantly focused on client health records and hospitals, emphasizing the need for broader research across healthcare services.

**Conclusions:**

With a clear mandate, dedicated time, and proper training, health personnel in champion roles can significantly contribute professional, technological, and personal competencies to facilitate technology adoption within healthcare services. The review finds that the concept of champions is a broad one and finds varied definitions of the champion role concept. This underscores the importance of describing organizational characteristics, and highlights areas for future research to enhance technology implementation strategies in different healthcare settings with support of a champion.

**Supplementary Information:**

The online version contains supplementary material available at 10.1186/s12913-024-10867-7.

## Background

Digital health technologies play a transformative role in healthcare service systems [1, 2]. The utilization of technology and digitalization is essential for ensuring patient safety, delivering high quality, cost-effective, and sustainable healthcare services [3, 4]. The implementation of technology in healthcare services is a complex process that demands systematic changes in roles, workflows, and service provision [5, 6].

The successful implementation of new technologies in healthcare services relies on the adaptability of health professionals [7, 8, 9]. Champions have been identified as a key factor in the successful implementation of technology among health personnel [10–12]. However, they have rarely been studied as an independent strategy; instead, they are often part of a broader array of strategies in implementation studies (e.g., Hudson [13], Gullslett and Bergmo [14]). Prior research has frequently focused on determining the presence or absence of champions [10, 12, 15], as well as investigating the characteristics of individuals assuming the champion role (e.g., George et al. [16], Shea and Belden [17]).

Recent reviews on champions [18, 19, 20] have studied their effects on adherence to guidelines, implementation of innovations and facilitation of evidence-based practice. While these reviews suggest that having champions yields positive effects, they underscore the importance for studies that offer detailed insights into the champion’s role concerning specific types of interventions.

There is limited understanding of the practical role requirements and the actual experiences of health personnel in performing the champion role in the context of technology implementation within healthcare services. Further, this knowledge is needed to guide future research on the practical, professional, and relational prerequisites for health personnel in this role and for organizations to successfully employ champions as a strategy in technology implementation processes.

This review seeks to synthesize the existing empirical knowledge concerning the experiences of those in the champion role and the perspectives of health personnel involved in technology implementation processes. The aim is to contribute valuable insights that enhance our understanding of practical role requirements, the execution of the champion role, and best practices in this domain.

The term of champions varies [10, 19] and there is a lack of explicit conceptualization of the term ‘champion’ in the implementation literature [12, 18]. Various terms for individuals with similar roles also exist in the literature, such as implementation leader, opinion leader, facilitator, change agent, superuser and facilitator. For the purpose of this study, we have adopted the terminology utilized in the recent review by Rigby, Redley and Hutchinson [21] collectively referring to these roles as ‘champions’. This review aims to explore the experiences of health personnel in their role as champions and the experiences of health personnel interacting with them in the implementation of technology in the healthcare services.

## Methods

Prior review studies on champions in healthcare services have employed various designs [10, 18, 19, 20]. In this review, we utilized a comprehensive mixed studies search to identify relevant empirical studies [22]. The search was conducted utilizing the Preferred Reporting Items for Systematic and Meta-Analysis (PRISMA) guidelines, ensuring a transparent and comprehensive overview that can be replicated or updated by others [23]. The study protocol is registered in PROSPERO (ID CRD42022335750), providing a more comprehensive description of the methods [24]. A systematic mixed studies review, examining research using diverse study designs, is well-suited for synthesizing existing knowledge and identifying gaps by harnessing the strengths of both qualitative and quantitative methods [22]. Our search encompassed qualitative, quantitative, and mixed methods design to capture experiences with the role of champions in technology implementation.

## Search strategy and study selection

### Search strategy

The first author, in collaboration with a librarian, developed the search strategy based on initial searches to identify appropriate terms and truncations that align with the eligibility criteria. The search was constructed utilizing a combination of MeSH terms and keywords related to technology, implementation, champion, and attitudes/experiences. Conducted in August/September 2022, the search encompassed four databases: Medline, Embase, CINAHL, and Scopus, with an updated search conducted in March 2023. The full search strategy for Medline is provided in Appendix 1. The searches in Embase, CINAHL and Scopus employed the same strategy, with adopted terms and phrases to meet the requirements of each respective database.

### Eligibility criteria

#### Inclusion

We included all empirical studies employing qualitative, quantitative, and mixed methods designs that detailed the experiences and/or attitudes of health personnel regarding the champions role in the implementation of technology in healthcare services. Articles in the English language published between 2012 and 2023 were considered. The selected studies involved technology implemented or adapted within healthcare services.

#### Exclusion

Conference abstract and review articles were excluded from consideration. Articles published prior 2012 were excluded as a result of the rapid development of technology, which could impact the experiences reported. Furthermore, articles involving surgical technology and pre-implementation studies were also excluded, as the focus was on capturing experiences and attitudes from the adoption and daily use of technology. The study also excluded articles that involved champions without clinical health care positions.

### Study selection

A total of 1629 studies were identified and downloaded from the selected databases, with Covidence [25] utilized as a software platform for screening. After removing 624 duplicate records, all team members collaborated to calibrate the screening process utilizing the eligibility criteria on the initial 50 studies. Subsequently, the remaining abstracts were independently screened by two researchers, blinded to each other, to ensure adherence to the eligibility criteria. Studies were included if the title and abstract included the term champion or its synonyms, along with technology in healthcare services, implementation, and health personnel’s experiences or attitudes. Any discrepancies were resolved through consensus among all team members. A total of 949 abstracts were excluded for not meeting this inclusion condition. During the initial search, 56 remaining studies underwent full-text screening, resulting in identification of 22 studies qualified for review.

**Fig. 1:**
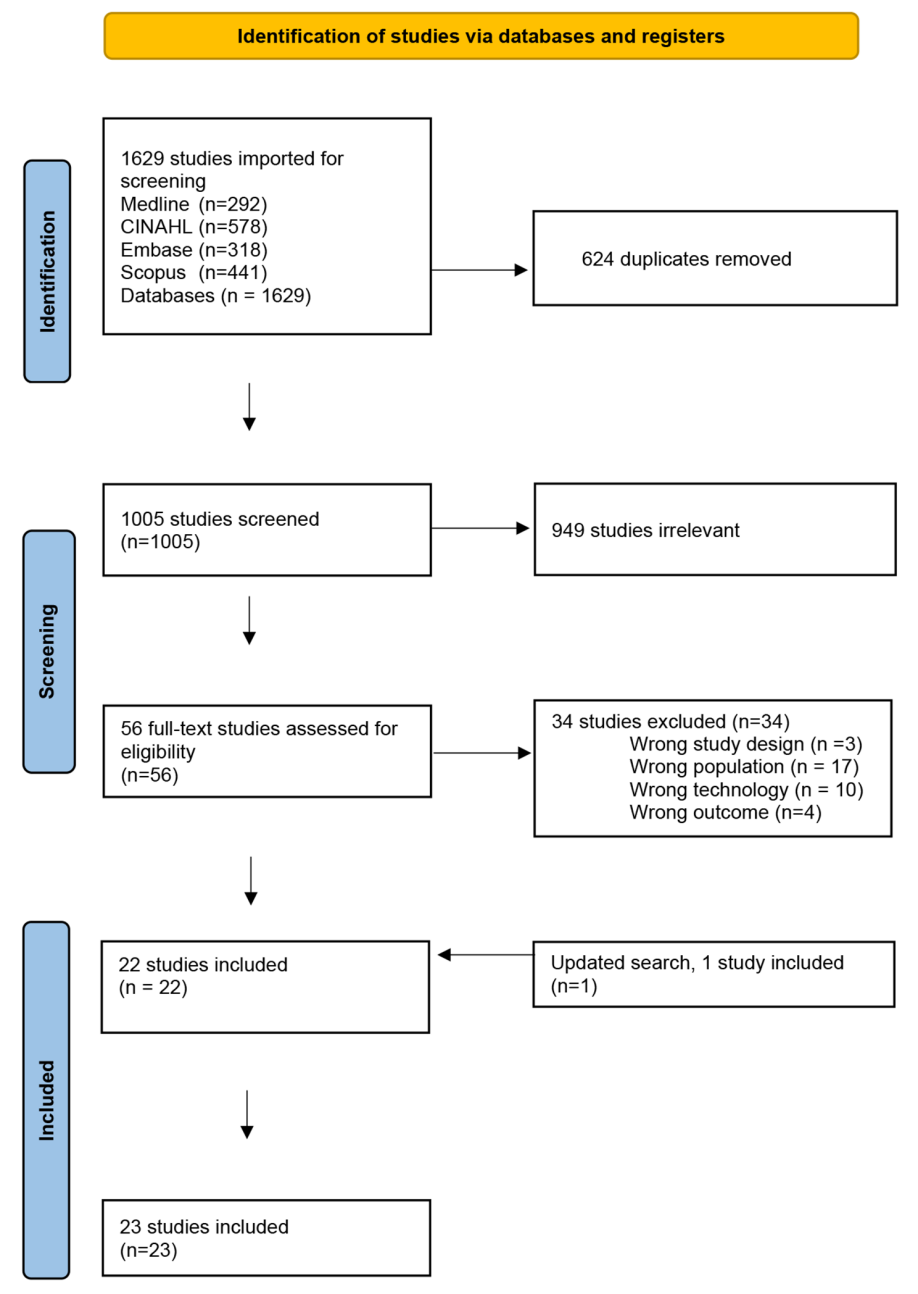
Flow Chart illustrating the study selection and screening process

In the updated search covering the period September 2022 to March 2023, 64 new studies were identified. Of these, 18 studies underwent full-text screening, and one study was included in our review. The total number of included studies is 23. The PRISMA flowchart (Fig. 1) illustrates the process.

### Data extraction

The research team developed an extraction form for the included studies utilizing an Excel spreadsheet. Following data extraction, the information included the Name of Author(s) Year of publication, Country/countries, Title of the article, Setting, Aim, Design, Participants, and Sample size of the studies, Technology utilized in healthcare services, name/title utilized to describe the Champion Role, how the studies were analyzed and details of Attitude/Experience with the role of champion. Data extraction was conducted by SP, and the results were deliberated in a workshop with the other researchers AB, and HE until a consensus was reached. Any discrepancies were resolved through discussions. The data extraction was categorized into three categories: qualitative, quantitative, and mixed methods, in preparation for quality appraisal.

### Quality appraisal

The MMAT [26] was employed to assess the quality of the 23 included studies. Specifically designed for mixed studies reviews, the MMAT allows for the appraisal of the methodological quality of studies falling into five categories. The studies in our review encompassed qualitative, quantitative descriptive, and mixed methods studies. The MMAT begins with two screening questions to confirm the empirical nature of this study. Subsequently, all studies were categorized by type and evaluated utilizing specific criteria based on their research methods, with ratings of ‘Yes,’ ‘No’ or ‘Can’t tell.’ The MMAT discourages overall scores in favor of providing a detailed explanation for each criterion. Consequently, we did not rely on the MMAT’s overall methodical quality scores and continued to include all 23 studies for our review. Two researchers independently scored the studies, and any discrepancies were discussed among all team members until a consensus was reached. The results of the MMAT assessments are provided in Appendix 2.

### Data synthesis

Based on discussions of this material, additional tables were formulated to present a comprehensive overview of the study characteristics categorized by study design, study settings, technology included, and descriptions/characteristics of the champion role. To capture attitudes and experiences associated with the champion role, the findings from the included studies were translated into narrative texts [22]. Subsequently, the reviewers worked collaboratively to conduct a thematic analysis, drawing inspiration from Braun and Clarke [27]. Throughout the synthesis process, multiple meetings were conducted to discern and define the emerging themes and subthemes.

The adopting of new technology in healthcare services can be perceived as both an event and a process. According to Iqbal [28], experience is defined as the knowledge and understanding gained after an event or the process of living through or undergoing an event. This review synthesizes existing empirical knowledge regarding the experiences of occupying the champion role, and the perspectives of health personnel interacting with champions in technology implementation processes.

## Results

### Study characteristics

The review encompassed a total of 23 studies, and an overview of these studies is presented in Table 1. Of these, fourteen studies employed a qualitative design, four had quantitative design, and five utilized a mixed method design. The geographical distribution revealed that the majority of studies were conducted in the USA (8), followed by Australia (5), England (4), Canada (2), Norway (2), Ireland (1), and Malaysia (1). In terms of settings, 11 studies were conducted in hospitals, five in primary health care, three in home-based care settings, and four in a mixed settings where two or more settings collaborated. Various technologies were employed across these studies, with client health records (7) and telemedicine (5) being the most frequently utilized. All studies included experiences from champions or health personnel collaborating with champions in their respective healthcare services. Only three studies had the champion role as a main objective [29, 30, 31]. The remaining studies described champions as one of the strategies in technology implementation processes, including 10 evaluation studies (including feasibility studies [32, 33, 34] and one cost-benefit study [30]).

**Table 1:** Study characteristics

**Study characteristics of qualitative studies (** ***n*** ** = 14)**							
Author/ Country	**Methodology and Methods**	**Sample**	**Aim**	**Setting**	**Technology**	**Role**	**Main results**
Bee et al. [35], UK	Qualitative design, semi-structured interviews. Descriptive implementation study	*N* = 18 18 therapists	To explore cognitive behavioral therapists’ narratives around T- CBT, with a view to identifying current and potential influences on its uptake and implementation in statutory mental health services.	Primary health care	Telemedicine	T-CBT-Champions	The local practice-based champion had the potential to influence other health professionals with enthusiasm and experiential knowledge.
Bennett-Levy et al. [41], Australia	Qualitative design, semi- structured interviews and reports. Program Evaluation study	*N* = 26 26 health professionals	To provide a qualitative evaluation of the impact of e-MH training plus follow-up consultation sessions with Aboriginal health providers.	Primary health care	Telemedicine	Tech-savvy champions	Tech savvy champions suggested ideas for practicing on the Stay Strong app.
Buckingham et al. [44], England	Qualitative design, guided discussions (individual and group). Program evaluation Study	*N* = 53 2 carers 7 patients 21 individuals practitioners 23 practitioners in group	To inform an online toolkit and training package (the Telerehab Toolkit) to assist the current and future health and social care workforce in conducting safe and effective remote physical assessments and consultations.	Mixed settings	Rehabilitation technologies	Digital champion	Digital champions are recommended to lead telerehabilitation and provide support to other staff members.
Chung et al. [45], Australia	Qualitative design, semi-structured interviews. Descriptive implementation study	*N* = 19 14 Cross-disciplinary clinicians and 5 service managers	The study sought to explore the perspectives of clinicians and service managers working in private mental healthcare regarding VR use, including potential implementation barriers and facilitators.	Hospital	Rehabilitation technologies	Opinion leader	Local opinion leaders are important for promoting, establishing credibility, and maintaining quality during the implementation period.
Fontaine et al. [36], USA	Qualitative design, semi-structured interviews. Descriptive implementation study	*N* = 31 31 administrative or clinical leaders	To identify the facilitators and barriers encountered by nine diverse primary care practices selected from the first 80 to achieve PCMH certification in Minnesota.	Home based care	Client Health records	Physician champions	Salary coverage for physicians and staff time that was devoted to PCMH development.
Gui et al. [29], USA	Qualitative design, email interviews. Descriptive implementation study	*N* = 45 45 Physician champions	To understand what strategies Physician champions must tackle challenges in their practices during the implementation and adoption processes of a EHR to be able to cultivate the best practices.	Mixed setting	Client Health records	Physician champion	Physician champions faced challenges, including inadequate training before start-up, insufficient elbow support after start-up, challenges in communication between the builders and suppliers’ company, and system design errors after start-up.
Hogan-Murphy et al. [46], Ireland	Qualitative design, semi-structured interviews. Descriptive implementation study	*N* = 23 23 Key stakeholders	To explore the perceptions of key stakeholders towards the facilitators and barriers to implementing electronic prescribing, robotic pharmacy systems, and automated medication storage and retrieval systems in public hospital settings.	Hospital	Prescription and Medication management	Clinical champions	Clinical champions at ward level promoted engagement. They are involved in planning and discussions, as well as discussing what will be functional in the ward.
Kolltveit et al. [37], Norway	Interpretive description, Qualitative design, focus group interviews. Descriptive implementation study	*N* = 34 24 registered nurses 5 clinical leaders 1 nurse assistant 2 podiatrists 2 physicians	To identify what health care professionals in distinct staff groups perceived as essential conditions for effective implementation of telemedicine as a new health care technology in diabetes foot care.	Mixed settings	Telemedicine	Telemedicine champion	A telemedicine champion was one of four key conditions for success. Those champions were described by the health personnel in the outpatient clinics as professional, updated, and engaged, and able to use the technology.
Moss et al. [47], USA	Qualitative design, semi-structured interviews. Descriptive implementation study	*N* = 17 17 participants. Fourteen providers, three site champions	To understand provider perspectives on VTE prophylaxis and facilitators and barriers to using the risk calculator.	Hospitals	Health care provider decision support	Physician champions and Site champions	Physician champions promoted the use of calculators. Site champions experienced larger facilities and private physicians were a barrier to calculator use.
Olsen et al. [42], USA	Qualitative design, open-ended questions in electronic survey format. Program evaluation study	N=?	To describe barriers and best practices learned when implementing EHR-based NDPP referral programs (National Diabetes Prevention Program) in two rural health care organizations.	Mixed setting	Client health records	Provider champions	Start small with one provider champion, and make sure to not exceed resources, the providers need time to master the new technology and the providers wants to be involved in the discussions and decisions.
Owens and Charles [32], England	Qualitative design, individual interviews and focus group interviews. Descriptive implementation study	*N* = 23 Focus groups with 14 team members, individual interviews with 7 clinicians and 2 service managers	To test and refine the intervention in situ, before proceeding to a full trial.	Primary health care	Behavior change technology (mHealth)	Committed participants, Product champions and Clinical champions	Early clinical champions may overestimate the clinician’s readiness for the intervention.
Salbach et al. [38], Canada	Qualitative design, individual interviews and focus group interviews. Descriptive implementation study	*N* = 40 33 Physical Therapists, 4 Professional Practice Leaders, 3 Professional Leaders. Program evaluation study	To examine how the contextual circumstances of acute care and of inpatient and outpatient rehabilitation practice settings influenced participants’ engagement with the toolkit and implementation strategy to effect practice change.	Hospital	Rehabilitation technology	Facilitators	Every site was asked to identify a facilitator. However, not all places had a facilitator, which resulted in no use of the strategy.
Yang et al. [43], USA	Qualitative design, Individual interviews. Descriptive implementation study	Reflections after 1000 video visits for 4 weeks. N =?	To describe Stanford Neurology Department’s transition of all subspecialty and general neurology patient consultations to stay at home video visits.	Hospital	Telemedicine	Physician champion	Physician champion (also named as key drivers) conducted 1:1 training session to approximately 50% of providers that was a significant factor for successful deployment. Physician champion gave virtually training to schedulers, including templates and checklists. Physician champion participated in daily huddles and answering emails and were available.
Yusof [48], Malaysia	Qualitative design, semi structured interviews, observation and document analysis. Program Evaluation study	*N* = 193 193 system users; 134 ICU nurses, 24 anesthetists, 23 perfusionists, 10 OT technicians, and 2 surgeons.	The purposes of the study are to (1) assess CCIS (Critical Care Information System) adoption level and issues in achieving its desired outcomes which subsequently affect healthcare delivery; (2) examine current CCIS implementation status; and (3) identify lessons from influential adoption factors to inform decision making.	Hospital	Client health records	IT nurse, super user, and IT savvy clinicians	Super users received inadequate training and were trained at the same time, as they were required to perform clinical tasks. Management’s support for a champion was lacking. Super users did not find evidence that the system made a significant difference. The super user experienced their own work, and the system created more IT-savvy employees.
**Study characteristics of quantitative studies (** ***n*** ** = 4)**							
Author/ Country	**Design**	**Sample**	**Aim**	**Setting**	**Technology**	**Role**	**Main results**
Bullard [30], USA	Quantitative design, descriptive study, case study. Cost-effective study	150+ recent graduated nurses	To explore the costs of EHR implementation with the nursing super-user role in a metropolitan, not-for-profit health care system.	Hospitals	Client Health records	Super-user	Reduced labor costs were associated with super-user staffing by 31.8%.
Dugstad et al. [49], Norway	Quantitative, cross-sectional descriptive design. Program Evaluation study	*N* = 98 79 Care providers 19 superusers	To explore healthcare providers’ evaluation of facilitators and barriers during implementation of WNCSs in residential care settings.	Home based care	Assistive technology	WNCS super user Digital transformation facilitator	The care providers could provide feedback regarding WNCS to their manager or a super user in a confident way (82%). There was a high degree of management engagement, and care providers reported feeling social support from the management and their colleagues (80%).
Rea et al. [50], USA	Quantitative descriptive design, electronic survey. Descriptive Implementation Study	*N* = 14 14 nurse champions	To investigate if use of a QI cloud-based software technology accessible on mobile devices causes differences in rates, causes differences in compliance with evidence-based CAUTI prevention practices, level of nurse champion satisfaction and identification of benefits and barriers and perceptions of adopting the technology.	Hospital	Health care provider decision support	Nurse champion	The nurse champion was responsible for quality improvement.
Schwarz et al. [39], Australia	Quantitative descriptive design, cross-sectional survey. Descriptive Implementation Study	*N* = 104 104 AHPs	To provide an overview of AHPs’ perceptions of EMR implementation across three sites (both regional and metropolitan), with a focus on identifying perceptions before, during, and after implementation in relation to subjective perceptions, barriers and facilitators and overall satisfaction.	Hospital	Client health records	Clinician change champion	The presence of a profession-specific clinician “change champion” was the most important factor in facilitating the implementation of electronic medical records among allied health personnel. The champion could provide practical and cultural support if needed. Additionally, 62% of respondents agreed that they received enough support during the implementation, while 8% disagreed.
**Study characteristics of mixed methods studies (** ***n*** ** = 5)**							
Author/ Country	**Design**	**Sample**	**Aim**	**Setting**	**Technology**	**Role**	**Main results**
Bail et al. [51], Australia	Mixed-methods case study, observations, individual interviews, surveys, focus group interviews and hospital admission data analysis. Descriptive Implementation Study	*N* = 152 20 patient interviews 33 patient surveys 48 clinician interviews 51 clinician surveys Admission data analysis	To investigate the implementation of a novel electronic bedside nursing chart in an acute hospital setting.	Hospital	Client Health records, electronic bedside nursing chart	Super-user	Six of eight trained nurse super-users were moved from the ward during the implementation period of four weeks, which was inconsistent with a trial implementation.
Helmer-Smith et al. [33], Canada	Multi-method approach, cross-sectional study, use data, close-out survey and focus group interviews. Program Evaluation study	*N* = 16 10 PCPs, 4 administrations, 2 nurse champions.	To explore the perceptions of key stakeholders towards the facilitators and barriers to implementing electronic prescribing (ePrescribing), robotic pharmacy systems, and automated medication storage and retrieval systems in public hospital settings.	Home based care	Telemedicine	Clinician champion	Clinical champions at the ward level promoted engagement. They are involved in planning and discussions, as well as discussing what will be functional in the ward.
Orchard et al. [34], Australia	Mixed Methods study. Cross sectional pilot study. Semi-structured interviews. Program Evaluation study	*N* = 989 972 patients screening, 17 health personnel interviews	To determine the feasibility of practice nurse screening in Australia during the flu-vaccination period.	Primary health care	Health care provider decision support	Practice manager	A practice champion was important for the success of the implementation since he/she led and enhanced it. The practice champion suggested that it is necessary to finance the setup and filing, as well as the expenses for the time spent by nurses during the screening process.
Stewart et al. [40], UK	Mixed-methods evaluation, online questionnaire, semi structured interviews and focus group interviews. Program Evaluation study	*N* = 63 63 General practice staff	To evaluate the rapid and reactive implementation of RAC in general practice in response to the COVID-19 pandemic, through the lens of eNPT, to identify factors that promoted or inhibited implementation, and identify the ‘work’ that is required for ‘normalization’ into routine clinical care.	Primary health care	Telemedicine	Practice champion	Identify a practice champion to lead the implementation, since no one usually takes the responsibility. A practice champion is often a GP who plays a strategic role in providing adaptive care. A practice champion was important for engaging other personnel.
Yuan et al. [31], USA	Mixed method design, comparative case study, observation, in-depth interviews and pre-/follow up surveys. Descriptive implementation study	*N* = 67 24 In-depth interviews superuser/non-superuser 43 pre-/follow up surveys	To provide insight that may help health care organizations better select, prepare, and support super users so that they can realize their potential for positive influence on the implementation of EHRs, and health information technology broadly.	Hospital	Client health records	Superuser	Volunteered superusers were proactive, explained through practical use and the logic behind it, used positive frameworks when discussing implementation procedure, and shared information about EHR. Department heads identified superusers who were tech-savvy; Designated superusers supported their employees on demand; they practically showed how the technology worked but did not explain the logic behind it. Designated superusers spoke neutrally about EHR and provided limited information.

Several studies underscored the importance of champions for successful implementation [29–31, 34–38, 40–43, 49]. Four studies specifically highlighted champions as a key factor for success [34, 36, 37, 43], and one study went further to describe champions as the most important factor for successful implementation [39]. Additionally, one study associated champions with reduced labor cost [30].

### Thin descriptions, yet clear expectations for technology champions’ role and -attributes

The analyses revealed that the concept of champions in studies pertaining to technology implementation in healthcare services varies, primarily as a result of the diversity of terms utilized to describe the role combined with short role descriptions. Nevertheless, the studies indicated clear expectations for the champion’s role and associated attributes.

#### The term champion

The term *champion* was expressed in 20 different forms across the 23 studies included in our review. Three studies utilized multiple terms within the same study [32, 47, 48] and 15 different authors [29, 32, 33, 35–37, 39–44, 46, 47, 50] employed the term with different compositions (Table 1). Furthermore, four authors utilized the term *Super user* [30, 31, 49, 51], while four authors employed the terms *Facilitator* [38], *IT clinician* [48], *Leader* [45], and *Manager* [34], each in combination with more specific terms (such as local opinion leaders, IT nurse, or practice manager).

Most studies associated champion roles with specific professions. In seven studies, the professional title was explicitly linked to the concept of champions, such as physician champions or clinical nurse champions, or through the strategic selection of specific professions [29, 33, 36, 40, 43, 47, 50]. Additionally, some studies did not specify professions, but utilized terms like *clinicians* [45] or *health professionals* [41].

All included articles portray the champion’s role as facilitating implementation and daily use of technology among staff. In four studies, the champion’s role was not elaborated beyond indicating that the individual holding the role is confident with an interest in technology [35, 41, 42, 44]. The champion’s role was explicitly examined in six studies [29–31, 33, 46, 50]. Furthermore, seven studies described the champion in both the methods and results [32, 36, 38, 47–49, 51]. In ten of the studies, champions were solely mentioned in the results [34, 35, 37, 39–45].

Eight studies provided a specific description or definition of the champion [29–32, 38, 48–50]. The champion’s role was described as involving training in the specific technology, being an expert on the technology, providing support and assisting peers when needed. In some instance, the champion had a role in leading the implementation [50], while in other situations, the champion operated as a mediator [48].

#### The champions tasks

In the included studies, the champion role encompassed two interrelated facilitators tasks: promoting the technology and supporting others in adopting the technology in their daily practice. Promoting the technology involved encouraging staff adaptation [32, 34, 35, 37, 40, 41, 49], generally described as being enthusiastic about the technology [32, 35, 37, 41, 48], influencing the attitudes and beliefs of colleagues [42, 45] and legitimizing the introduction of the technology [42, 46, 48]. Supporting others in technology adaption involved training and teaching [31, 35, 38, 40, 51], as well as providing technical support [30, 31, 39, 43, 49] and social support [49]. Only four studies reported that the champions received their own training to enable them able to support their colleagues [30, 31, 39, 48]. Furthermore, eight studies [32, 34, 38, 40, 48–51], specified that the champion role included leadership and management responsibilities, mentioning tasks such as planning, organizing, coordinating, and mediating technology adaption without providing further details.

#### Desirable champion attributes

To effectively fulfill their role, champions should ideally possess clinical expertise and experience [29, 35, 38, 40, 48], stay professionally updated [37, 48], and possess knowledge of the organization and workflows [29, 34, 46]. They should have the ability to understand and communicate effectively with healthcare personnel [31, 32, 46, 49] and be proficient in IT language [51]. Moreover, champions should demonstrate a general technological interest and competence, and competence, along with specific knowledge of the technology to be implemented [32, 37, 49]. It is also emphasized that they should command formal and/or informal respect and authority in the organization [36, 45], be accessible to others [39, 43], possess leadership qualities [34, 37, 38, 46], and understand and balance the needs of stakeholders [43]. Lastly, the champions should be enthusiastic promoters of the technology, engaging and supporting others [31–34, 37, 39–41, 43, 49], while also effectively coping with cultural resistance to change [31, 46].

### Anchoring and recruiting for the champion role

The champions were organized differently within services, holding various positions in the organizations, and being recruited for the role in different ways.

#### Anchoring the champion role

The champion’s role is primarily anchored at two levels: the management level and/or the clinical level, with two studies having champions at both levels [34, 49]. Those working with the management actively participated in the planning of the technology implementation [29, 36, 40, 41, 45]. Serving as advisors to management, they leveraged their clinical knowledge to guide the implementation in alignment with the necessities and possibilities of daily work routines in the clinics. Champions in this capacity experienced having a clear formal position that enabled them to fulfil their role effectively [29, 40]. Moreover, these champions served as bridge builders between the management and department levels [36, 45], ensuring the necessary flow of information in both directions.

Champions anchored at the clinic level played a pivotal role in the practical implementation and facilitation of the daily use of technology [31, 33, 35, 37, 38, 43, 48, 51]. Additionally, these champions actively participated in meetings with senior management to discuss the technology and its implementation in the clinic. This position conferred potential influence over health personnel [33, 35]. Champions at the clinic level facilitated collaboration between employees, management, and suppliers [48]. Fontaine et al. [36] identified respected champions at the clinical level, possessing authority and formal support from all leadership levels, as the most important factor for success.

Only one study reported that the champions received additional compensation for their role [36], while another study mentioned champions having dedicated time to fulfil their role [46]. The remaining studies did not provide this information.

#### Recruiting for the role as champion

Several studies have reported different experiences regarding the management’s selection of champions. A study highlighted the distinctions between a volunteered role and an appointed champion’s role [31]. Some studies underscored that appointed champions were chosen based on technological expertise and skills [41, 48, 51]. Moreover, the selection criteria included champions’ interest in the specific technology [42] or experiential skills [40]. The remaining studies did not provide this information.

While the champion role was most frequently held by health personnel with clinical experience, one study deviated by hiring 150 newly qualified nurses as champions [30] for a large-scale implementation of an Electronic Health Record (EHR). Opting for clinical novices assisted in reducing implementation costs, as it avoided disrupting daily tasks and interfering with daily operations. According to Bullard [30], these super-user nurses became highly sought after post-implementation as a result of their technological confidence and competence.

### Reported experiences of champions and health personnel

Drawing from the experiences of both champions and health personnel, it is essential for a champion to possess a combination of general knowledge and specific champion characteristics. Furthermore, champions are required to collaborate with individuals both within and outside the organization. The subsequent paragraphs delineate these experiences, categorizing them into four subsets: champions’ contextual knowledge and expertise, preferred performance of the champion role, recognizing that a champion alone is insufficient, and distinguishing between reactive and proactive champions.

#### Champions’ contextual knowledge and know-how

Health personnel with experience interacting with champions emphasized that a champion must be familiar with the department and its daily work routines [35, 40]. Knowledge of the department’s daily routines made it easier for champions to facilitate the adaptation of technology. However, there was a divergence of opinions on whether champions were required to possess extensive clinical experience to fulfil their role. In most studies, having an experienced and competent clinician as a champion instilled a sense of confidence among health personnel. Conversely, Bullard’s study [30] exhibited that health personnel were satisfied with newly qualified nurses in the role of champion, despite their initial skepticism.

It is a generally expected that champions should possess technological knowledge beyond that of other health professionals [37, 41]. Some health personnel perceived the champions as uncritical promoters of technology, with the impression that health personnel were being compelled to utilize technology [46]. Champions could also overestimate the readiness of health personnel to implement a technology, especially during the early phases of the implementation process [32]. Regardless of whether the champion is at the management level or the clinic level, champions themselves have acknowledged the importance of providing time and space for innovation. Moreover, the recruitment of champions should span all levels of the organization [34, 46]. Furthermore, champions must be familiar with daily work routines, work tools, and work surfaces [38, 40, 43].

#### Preferable performance of the champion role

The studies identified several preferable characteristics of successful champions. Health personnel favored champions utilizing positive words when discussing technology and exhibiting positive attitudes while facilitating and adapting it [33, 34, 37, 38, 41, 46]. Additionally, champions who were enthusiastic and engaging were considered good role models for the adoption of technology. Successful champions were perceived as knowledgeable and adept problem solvers who motivated and supported health personnel [41, 43, 44, 48]. They were also valued for being available and responding promptly when contacted [42]. Health professionals noted that champions perceived as competent garnered respect in the organization [40]. Moreover, some health personnel felt that some certain champions wielded a greater influence based on how they encouraged the use of the system [48]. It was also emphasized that health personnel needed to feel it was safe to provide feedback to champions, especially when encountering difficulties or uncertainties [49].

#### A champion is not enough

The role of champions proved to be more demanding than expected [29, 31, 38], involving tasks such as handling an overwhelming number of questions or actively participating in the installation process to ensure the technology functions effectively in the department [29]. Regardless of the organizational characteristics or the champion’s profile, appointing the champion as a “solo implementation agent” is deemed unsuitable. If the organization begins with one champion, it is recommended that this individual promptly recruits others into the role [42].

Health personnel, reliant on champions’ expertise, found it beneficial to have champions in all departments, and these champions had to be actively engaged in day-to-day operations [31, 33, 34, 37]. Champions themselves also noted that health personnel increased their technological expertise through their role as champions in the department [39].

Furthermore, the successful implementation of technology requires the collaboration of various professions and support functions, a task that cannot be solely addressed by a champion [29, 43, 48]. In Orchard et. al.‘s study [34], champions explicitly emphasized the necessity of support from other personnel in the organization, such as those responsible for the technical aspects and archiving routines, to provide essential assistance.

According to health personnel, the role of champions is vulnerable in case they become sick or leave their position [42, 51]. In some of the included studies, only one or a few hold the position of champion [37, 38, 42, 48]. Two studies observed that their implementations were not completed because champions left or reassigned for various reasons [32, 51]. The health professionals in the study by Owens and Charles [32] expressed that champions must be replaced in such cases. Further, the study of Olsen et al., 2021 [42] highlights the need for quicky building a champion network within the organization.

#### Reactive and proactive champions

Health personnel and champions alike noted that champions played both a reactive and proactive role. The proactive role entailed facilitating measures such as training and coordination [31–34, 37, 39–41, 43, 48, 49] as initiatives to generate enthusiasm for the technology [31–35, 37, 39–41, 43, 49]. On the other hand, the reactive role entailed hands-on support and troubleshooting [30, 31, 39, 43, 49].

In a study presenting experiences from both health personnel and champions, Yuan et al. [31] found that personnel observed differences in the assistance provided by appointed and self-chosen champions. Appointed champions demonstrated the technology, answered questions from health personnel, but quickly lost patience and track of employees who had received training [31]. Health personnel perceived that self-chosen champions were proactive and well-prepared to facilitate the utilization of technology, communicating with the staff as a group and being more competent in utilizing the technology in daily practice [31]. Health personnel also noted that volunteer champions were supportive, positive, and proactive in promoting the technology, whereas appointed champions acted on request and had a more reactive approach [31].

## Discussion

This review underscores the breadth of the concept of champion and the significant variation in the champion’s role in implementation of technology in healthcare services. This finding supports the results from previous reviews [10, 18, 19, 20]. The majority of studies meeting our inclusion criteria did not specifically focus on the experiences of champions and health personnel regarding the champion role, with the exception of studies by Bullard [30], Gui et al. [29], Helmer-Smith et al. [33], Hogan-Murphy et al. [46], Rea et al. [50], and Yuan et al. [31].

The 23 studies encompassed in this review utilized 20 different terms for the champion role. In most studies, the champion’s role was briefly described in terms of the duties it entailed or should entail. This may be linked to the fact that the role of champions was not the primary focus of the study, but rather one of the strategies in the implementation process being investigated. This result reinforces the conclusions drawn by Miech et al. [10] and Shea et al. [12] regarding the lack of united understandings of the concept. Furthermore, in Santos et al.‘s [19] review, champions were only operationalized through presence or absence in 71.4% of the included studies. However, our review finds that there is a consistent and shared understanding that champions should promote and support technology implementation.

Several studies advocate for champions as an effective and recommended strategy for implementing technology [30, 31, 33, 34, 35, 36, 37, 38, 39, 40, 42, 43, 45, 46]. However, we identified that few studies exclusively explore health personnel`s experiences within the champion role when implementing technology in healthcare services.

This suggests a general lack of information essential for understanding the pros, cons, and prerequisites for champions as a strategy within this field of knowledge. However, this review identifies, on a general basis, the types of support and structures required for champions to perform their role successfully from the perspectives of health personnel, contributing to Shea’s conceptual model [12].

Regarding the organization of the role, this review identified champions holding both formal appointed and informal roles, working in management or clinical settings, being recruited for their clinical and/or technological expertise, and either volunteering or being hired with specific benefits for the role. Regardless of these variations, anchoring the role is crucial for both the individuals holding the champion role and the health personnel interacting with them. Anchoring, in this context, is associated with the clarity of the role’s content and a match between role expectations and opportunities for fulfilment. Furthermore, the role should be valued by the management, preferably through dedicated time and/or salary support [34, 36, 46]. Additionally, our findings indicate that relying on a “solo champion” is vulnerable to issues such as illness, turnover, excessive workload, and individual champion performance [32, 37]. Based on these insights, it appears preferable to appoint multiple champions, with roles at both management and clinical levels [33].

Some studies have explored the selection of champions and its impact on role performance, revealing diverse experiences [30, 31]. Notably, Bullard [30], stands out for emphasizing long clinical experience, and hiring newly trained nurses as superusers to facilitate the use of electronic health records. Despite facing initial reluctance, these newly trained nurses gradually succeeded in their roles. This underscores the importance of considering contextual factors in the champion selection [30, 52]. In Bullard’s study [30], the collaboration between newly trained nurses as digital natives and clinical experienced health personnel proved beneficial, highlighting the need to align champion selection with the organization’s needs based on personal characteristics. This finding aligns with Melkas et al.‘s [9] argument that implementing technology requires a deeper understanding of users, access to contextual know-how, and health personnel’s tacit knowledge.

To meet role expectations and effectively leverage their professional and technological expertise, champions should embody personal qualities such as the ability to engage others, take a leadership role, be accessible, supportive, and communicate clearly. These qualities align with the key attributes for change in healthcare champions described by Bonawitz et al. [15]. These attributes include influence, ownership, physical presence, persuasiveness, grit, and a participative leadership style (p.5). These findings suggest that the active performance of the role, beyond mere presence, is crucial for champions to be a successful strategy in technology implementation. Moreover, the recruitment process is not inconsequential. Identifying the right person for the role and providing them with adequate training, organizational support, and dedicated time to fulfill their responsibilities emerge as an important factor based on the insights from champions and health personnel.

### Strengths and limitations

While this study benefits from identifying various terms associated with the role of champions, it acknowledges the possibility of missing some studies as a result of diverse descriptions of the role. Nonetheless, a notable strength of the study lies in its specific focus on the health personnel’s experiences in holding the champion role and the broader experiences of health personnel concerning champions in technology implementation within healthcare services. This approach contributes valuable insights into the characteristics of experiences and attitudes toward the role of champions in implementing technology. Lastly, the study emphasizes the relationship between the experiences with the champion role and the organizational setting’s characteristics.

The champion role was frequently inadequately defined [30, 33–37, 39, 41–47, 51], aligning with previous reviews [17, 19, 21]. As indicated by van Laere and Aggestam [52], this lack of clarity complicates the identification and comparison of champions across studies. Studies that lacking a distinct definition of the champion’s role were consequently excluded. Only studies written in English were included, introducing the possibility of overlooking relevant studies based on our chosen terms for identifying the champion’s role. Most of the included studies focused on technology implementation in a general context, with champions being just one of several measures. This approach resulted in scant descriptions, as champions were often discussed in the results, discussion, or implications sections rather than being the central focus of the research.

As highlighted by Hall et al. [18]., methodological issues and inadequate reporting in studies of the champion role create challenges for conducting high-quality reviews, introducing uncertainty around the findings. We have adopted a similar approach to Santos et al. [19], including all studies even when some issues were identified during the quality assessment. Our review shares the same limitations as previous review by Santos et al. [19] on the champion role.

### Practical implications, policy, and future research

The findings emphasize the significance of the relationship between experiences with the champion role and characteristics of organizational settings as crucial factors for success in the champion role. Clear anchoring of the role within the organization is vital and may impact routines, workflows, staffing, and budgets. Despite limited evidence on the experience of the champion’s role, volunteering, hiring newly graduated health personnel, and appointing more than one champion are identified as facilitators of technology implementation. This study underscores the need for future empirical research including clear descriptions of the champion roles, details on study settings and the technologies to be adopted. This will enable the determination of outcomes and success factors in holding champions in technology implementation processes, transferability of knowledge between contexts and technologies as well as enhance the comparability of studies. Furthermore, there is a need for studies to explore experiences with the champion role, preferably from the perspective of multiple stakeholders, as well as focus on the champion role within various healthcare settings.

## Conclusion

This study emphasizes that champions can hold significant positions when provided with a clear mandate, dedicated time, and training, contributing their professional, technological, and personal competencies to expedite technology adoption within services. It appears to be an advantage if the health personnel volunteer or apply for the role to facilitate engaged and proactive champions. The implementation of technology in healthcare services demands efforts from the entire service, and the experiences highlighted in this review exhibits that champions can play an important role. Consequently, empirical studies dedicated to the champion role, employing robust designs based current knowledge, are still needed to provide solid understanding of how champions can be a successful initiative when implementing technology in healthcare services.

### Electronic supplementary material

Below is the link to the electronic supplementary material.

Supplementary Material 1

Supplementary Material 2

## Data Availability

This review relies exclusively on previously published studies. The datasets supporting the conclusions of this article are included within the article and its supplementary files: Description and characteristics of included studies in Table 1, Study characteristics. The search strategy is provided in Appendix 1, and the Critical Appraisal Summary of included studies utilizing MMAT is presented in Appendix 2.

## Abbreviations

EHR: Electronic Health Record
IOF: Implementation Outcomes Framework
PRISMA: Preferred Reporting Items for Systematics and Meta-Analysis

## References

[1] Meskó B, Drobni Z, Bényei É, Gergely B, Győrffy Z (2017). Digital health is a cultural transformation of traditional healthcare. mHealth.

[2] Pérez Sust P, Solans O, Fajardo JC, Medina Peralta M, Rodenas P, Gabaldà J (2020). Turning the crisis into an opportunity: Digital health strategies deployed during the COVID-19 outbreak. JMIR Public Health Surveill.

[3] Alotaibi YK, Federico F (2017). The impact of health information technology on patient safety. Saudi MedJ.

[4] Kuoppamäki S (2021). The application and deployment of welfare technology in Swedish municipal care: a qualitative study of procurement practices among municipal actors. BMC Health Serv Res.

[5] Kraus S, Schiavone F, Pluzhnikova A, Invernizzi AC (2021). Digital transformation in healthcare: analyzing the current state-of-research. J Bus Res.

[6] Frennert S (2020). Approaches to welfare technology in municipal eldercare. JTechnolHum.

[7] Konttila J, Siira H, Kyngäs H, Lahtinen M, Elo S, Kääriäinen M (2019). Healthcare professionals’ competence in digitalisation: a systematic review. Clin Nurs.

[8] Jacob C, Sanchez-Vazquez A, Ivory C (2020). Social, organizational, and technological factors impacting clinicians’ adoption of mobile health tools: systematic literature review. JMIR mHealth uHealth.

[9] Melkas H, Hennala L, Pekkarinen S, Kyrki V (2020). Impacts of robot implementation on care personnel and clients in elderly-care institutions. Int J Med Inf.

[10] Miech EJ, Rattray NA, Flanagan ME, Damschroder L, Schmid AA, Damush TM. Inside help: an integrative review of champions in healthcare-related implementation. SAGE Open Med. 2018;6. 10.1177/2050312118773261. 10.1177/2050312118773261 PMC5960847 29796266

[11] Foong HF, Kyaw BM, Upton Z, Tudor Car L (2020). Facilitators and barriers of using digital technology for the management of diabetic foot ulcers: a qualitative systematic review. Int Wound J.

[12] Shea CM. A conceptual model to guide research on the activities and effects of innovation champions. Implement Res Pract. 2021;2. 10.1177/2633489521990443. 10.1177/2633489521990443 PMC8445003 34541541

[13] Hudson D (2023). Physician engagement strategies in health information system implementations. Healthc Manage Forum.

[14] Gullslett MK, Strand Bergmo T (2022). Implementation of E-prescription for multidose dispensed drugs: qualitative study of general practitioners’’ experiences. JMIR HumFactors.

[15] Bonawitz K, Wetmore M, Heisler M, Dalton VK, Damschroder LJ, Forman J (2020). Champions in context: which attributes matter for change efforts in healthcare?. Implement Sci.

[16] George ER, Sabin LL, Elliott PA, Wolff JA, Osani MC, McSwiggan Hong J, et al. Examining health care champions: a mixed-methods study exploring self and peer perspectives of champions. Implement Res Pract. 2022;3. 10.1177/26334895221077880. 10.1177/26334895221077880 PMC9924235 37091082

[17] Shea CM, Belden CM (2016). What is the extent of research on the characteristics, behaviors, and impacts of health information technology champions? A scoping review. BMC Med Inf Decis Mak.

[18] Hall AM, Flodgren GM, Richmond HL, Welsh S, Thompson JY, Furlong BM, Sherriff A (2021). Champions for improved adherence to guidelines in long-term care homes: a systematic review. Implement Sci Commun.

[19] Santos WJ, Graham ID, Lalonde M, Demery Varin M, Squires JE (2022). The effectiveness of champions in implementing innovations in health care: a systematic review. Implement Sci Commun.

[20] Wood K, Giannopoulos V, Louie E, Baillie A, Uribe G, Lee KS, Haber PS, Morley KC (2020). The role of clinical champions in facilitating the use of evidence-based practice in drug and alcohol and mental health settings: a systematic review. Implement Res Pract.

[21] Rigby K, Redley B, Hutchinson AM. Change agent’s role in facilitating use of technology in residential aged care: a systematic review. Int J Med Informatics. 2023;105216. 10.1016/j.ijmedinf.2023.105216. 10.1016/j.ijmedinf.2023.105216 37734272

[22] Pluye P, Hong QN (2014). Combining the power of stories and the power of numbers: mixed methods research and mixed studies reviews. Annu Rev Public Health.

[23] Page MJ, McKenzie JE, Bossuyt PM, Boutron I, Hoffmann TC, Mulrow CD (2021). The PRISMA 2020 statement: an updated guideline for reporting systematic reviews. BMJ.

[24] Pettersen S, Berg A, Eide H. Experiences and attitudes to the role of champions in implementation of technology in health services. A systematic review. PROSPERO. 2022. https://www.crd.york.ac.uk/prospero/display_record.php?ID=CRD42022335750. Accessed [15 Feb 2023].

[25] Covidence. Better Syst Rev Manag. https://www.covidence.org/. 2023;26.

[26] Hong QN, Fàbregues S, Bartlett G, Boardman F, Cargo M, Dagenais P (2018). The mixed methods Appraisal Tool (MMAT) version 2018 for information professionals and researchers. Educ Inf.

[27] Braun V, Clarke V. Thematic analysis: a practical guide. 1st ed. SAGE; 2022.

[28] Iqbal MP, Manias E, Mimmo L, Mears S, Jack B, Hay L, Harrison R (2020). Clinicians’ experience of providing care: a rapid review. BMC Health Serv Res.

[29] Gui X, Chen Y, Zhou X, Reynolds TL, Zheng K, Hanauer DA (2020). Physician champions’ perspectives and practices on electronic health records implementation: challenges and strategies. JAMIA open.

[30] Bullard KL (2016). Cost effective staffing for an EHR implementation. Nurs Econ.

[31] Yuan CT, Bradley EH, Nembhard IM (2015). A mixed methods study of how clinician ‘super users’ influence others during the implementation of electronic health records. BMC Med Inf Decis Mak.

[32] Owens C, Charles N (2016). Implementation of a text-messaging intervention for adolescents who self-harm (TeenTEXT): a feasibility study using normalisation process theory. Child Adolesc Psychiatry Ment Health.

[33] Helmer-Smith M, Fung C, Afkham A, Crowe L, Gazarin M, Keely E (2020). The feasibility of using electronic consultation in long-term care homes. JAm Med Dir Assoc.

[34] Orchard J, Lowres N, Freedman SB, Ladak L, Lee W, Zwar N (2016). Screening for atrial fibrillation during influenza vaccinations by primary care nurses using a smartphone electrocardiograph (iECG): a feasibility study. Eur J Prev Cardiol.

[35] Bee P, Lovell K, Airnes Z, Pruszynska A (2016). Embedding telephone therapy in statutory mental health services: a qualitative, theory-driven analysis. BMC Psychiatry.

[36] Fontaine P, Whitebird R, Solberg LI, Tillema J, Smithson A, Crabtree BF (2015). Minnesota’s early experience with medical home implementation: viewpoints from the front lines. J Gen Intern Med.

[37] Kolltveit B-CH, Gjengedal E, Graue M, Iversen MM, Thorne S, Kirkevold M (2017). Conditions for success in introducing telemedicine in diabetes foot care: a qualitative inquiry. BMC Nurs.

[38] Salbach NM, McDonald A, MacKay-Lyons M, Bulmer B, Howe JA, Bayley MT (2021). Experiences of physical therapists and professional leaders with implementing a toolkit to advance walking assessment poststroke: a realist evaluation. Phys Ther.

[39] Schwarz M, Coccetti A, Draheim M, Gordon G (2020). Perceptions of allied health staff of the implementation of an integrated electronic medical record across regional and metropolitan settings. Aust Health Rev.

[40] Stewart J, McCorry N, Reid H, Hart N, Kee F (2022). Implementation of remote asthma consulting in general practice in response to the COVID-19 pandemic: an evaluation using extended normalisation process theory. BJGP Open.

[41] Bennett-Levy J, Singer J, DuBois S, Hyde K (2017). Translating mental health into practice: what are the barriers and enablers to e-mental health implementation by aboriginal and Torres Strait Islander health professionals? JMed. Internet Res.

[42] Olsen J, Peterson S, Stevens A (2021). Implementing electronic health record-based National Diabetes Prevention Program referrals in a rural county. Public Health Nurs (Boston Mass).

[43] Yang L, Brown-Johnson CG, Miller-Kuhlmann R, Kling SMR, Saliba-Gustafsson EA, Shaw JG (2020). Accelerated launch of video visits in ambulatory neurology during COVID-19: key lessons from the Stanford experience. Neurology.

[44] Buckingham SA, Sein K, Anil K, Demain S, Gunn H, Jones RB (2022). Telerehabilitation for physical disabilities and movement impairment: a service evaluation in South West England. JEval Clin Pract.

[45] Chung OS, Robinson T, Johnson AM, Dowling NL, Ng CH, Yücel M (2022). Implementation of therapeutic virtual reality into psychiatric care: clinicians’ and service managers’’ perspectives. Front Psychiatry.

[46] Hogan-Murphy D, Stewart D, Tonna A, Strath A, Cunningham S (2021). Use of normalization process theory to explore key stakeholders’ perceptions of the facilitators and barriers to implementing electronic systems for medicines management in hospital settings. Res SocialAdm Pharm.

[47] Moss SR, Martinez KA, Nathan C, Pfoh ER, Rothberg MB (2022). Physicians’ views on utilization of an electronic health record-embedded calculator to assess risk for venous thromboembolism among medical inpatients: a qualitative study. TH Open.

[48] Yusof MM (2015). A case study evaluation of a critical Care Information System adoption using the socio-technical and fit approach. Int J Med Inf.

[49] Dugstad J, Sundling V, Nilsen ER, Eide H (2020). Nursing staff’s evaluation of facilitators and barriers during implementation of wireless nurse call systems in residential care facilities. A cross-sectional study. BMC Health Serv Res.

[50] Rea K, Le-Jenkins U, Rutledge C (2018). A technology intervention for nurses engaged in preventing catheter-associated urinary tract infections. Comput Inf Nurs.

[51] Bail K, Davey R, Currie M, Gibson J, Merrick E, Redley B (2020). Implementation pilot of a novel electronic bedside nursing chart: a mixed-methods case study. Aust Health Rev.

[52] van Laere J, Aggestam L (2016). Understanding champion behaviour in a health-care information system development project – how multiple champions and champion behaviours build a coherent whole. Eur J Inf Syst.

