# Supplementary material for: The role of champions in the implementation of technology in healthcare services: a systematic mixed studies review
Source: BMC Health Serv Res. 2024 Apr 11;24:456. doi: 10.1186/s12913-024-10867-7 (PMC11007964; doi:10.1186/s12913-024-10867-7)
Supplement: Supplementary file 1 — Supplementary Material 1 [file 12913_2024_10867_MOESM1_ESM.docx]

Appendix 1 Medline.

( ( TITLE-ABS-KEY ( "Electronic Health Record" OR "Electronic Health Records" OR "EHR" OR "EPR" OR "EPHR" OR "Wireless Technology" OR "Wireless Technologies" OR "Medication System" OR "Medication systems" OR "Wearable Electronic Device*" OR "Wearable sensor*" OR ( ( "digital or electronic) W/1 (sensor* or device* or monitor*" ) ) OR "Computer" OR "Computers" OR "ipad*" OR "Telemedicine" OR "Telehealth" OR "E-health" OR "ehealth" OR "Mobile health" OR "M-health" OR "mhealth" OR "Robotic" OR "Robotics" OR ( ( "welfare" OR "assist*" ) W/1 ( "technolog*" ) ) OR "Technology" OR "technologies" OR ( "Mobile" W/1 ( "app" OR "apps" OR "application*" ) ) OR "cellphone*" OR "cell phone*" OR "mobile phone*" OR "smartphone*" OR "smart phone*" OR "smart home*" OR "remote consultation" OR "sensor*" OR "Geographic Information System*" ) )

AND

( TITLE-ABS-KEY ( "implement*" OR "utiliz*" OR "improv*" OR "introduc*" OR "init*" OR "realis*" OR "rectif*" OR "innovat*" OR "appl*" OR "diffus*" ) ) )

AND

( TITLE-ABS-KEY ( "Champion*" OR "Superuser*" OR "Super-user*" OR "Super user*" OR "key user*" OR "expert user*" OR "ambassador*" OR "innovator" OR "innovators" OR "change agent*" OR "digital agent*" OR "health resource*" OR "facilitator*" ) )

AND

( TITLE-ABS-KEY ( ( ( "attitude*" OR "experience*" OR "view*" OR "perception*" OR "perspective*" OR "comprehension*" ) W/1 ( "health personnel" OR "nurse*" OR "physical therapist*" OR "occupational therapist*" OR "physician*" OR "pharmacist*" ) ) ) )
