# Supplementary material for: The role of champions in the implementation of technology in healthcare services: a systematic mixed studies review
Source: BMC Health Serv Res. 2024 Apr 11;24:456. doi: 10.1186/s12913-024-10867-7 (PMC11007964; doi:10.1186/s12913-024-10867-7)
Supplement: Supplementary file 2 — Supplementary Material 2 [file 12913_2024_10867_MOESM2_ESM.docx]

Appendix 2. Critical appraisal summary of included studies using MMAT

| Author/year | S1. Are there clear research questions? | S2. Do the collected data allow to address the research questions? | Qualitative studies^1^ | | | | | Quantitative descriptive studies^2^ | | | | | Mixed methods studies^3^ | | | | |
| --- | --- | --- | --- | --- | --- | --- | --- | --- | --- | --- | --- | --- | --- | --- | --- | --- | --- |
|  |  |  | 1.1 | 1.2 | 1.3 | 1.4 | 1.5 | 4.1 | 4.2 | 4.3 | 4.4 | 4.5 | 5.1 | 5.2 | 5.3 | 5.4 | 5.5 |
| Bail, et al., 2020 | Yes | Yes |  |  |  |  |  |  |  |  |  |  | Yes | Yes | Yes | Can’t tell | Yes |
| Bee, et al., 2016 | Yes | Yes | Yes | Yes | Yes | Yes | Yes |  |  |  |  |  |  |  |  |  |  |
| Bennett-Levy, et al., 2017 | Yes | Yes | Yes | Yes | Yes | Yes | Yes |  |  |  |  |  |  |  |  |  |  |
| Buckingham et al., 2022 | Yes | Yes | Yes | Yes | Yes | Yes | Yes |  |  |  |  |  |  |  |  |  |  |
| Bullard, 2016 | Yes | Yes |  |  |  |  |  | Yes | Yes | Yes | Yes | Yes |  |  |  |  |  |
| Chung et al., 2022 | Yes | Yes | Yes | Yes | Yes | Yes | Yes |  |  |  |  |  |  |  |  |  |  |
| Dugstad et al., 2020 | Yes | Yes |  |  |  |  |  | Yes | Yes | Yes | Yes | Yes |  |  |  |  |  |
| Fontaine et al., 2015 | Yes | Yes | Yes | Yes | Yes | Yes | Yes |  |  |  |  |  |  |  |  |  |  |
| Gui et al., 2020 | Yes | Yes | Yes | Yes | Yes | Yes | Yes |  |  |  |  |  |  |  |  |  |  |
| Helmer-Smith et al., 2020 | Yes | Yes |  |  |  |  |  |  |  |  |  |  | Yes | Yes | Can’t tell | Can’t tell | Yes |
| Hogan-Murphy et al., 2021 | Yes | Yes | Yes | Yes | Yes | Yes | Yes |  |  |  |  |  |  |  |  |  |  |
| Kolltveit et al., 2017 | Yes | Yes | Yes | Yes | Yes | Yes | Yes |  |  |  |  |  |  |  |  |  |  |
| Moss et al., 2022 | Yes | Yes | Yes | Yes | Yes | Yes | Yes |  |  |  |  |  |  |  |  |  |  |
| Olsen et al., 2021 | Yes | Yes | Yes | Yes | Yes | Yes | Yes |  |  |  |  |  |  |  |  |  |  |
| Orchard et al., 2016 | Yes | Yes |  |  |  |  |  |  |  |  |  |  | Yes | Yes | Yes | No | Can’t tell |
| Owens and Charles 2016 | Yes | Yes | Yes | Yes | Yes | Yes | Yes |  |  |  |  |  |  |  |  |  |  |
| Rea et al. 2018 | Yes | Yes |  |  |  |  |  | Yes | Yes | Yes | Yes | Yes |  |  |  |  |  |
| Salbach et al., 2021 | Yes | Yes | Yes | Yes | Yes | Yes | Yes |  |  |  |  |  |  |  |  |  |  |
| Schwarz et al., 2020 | Yes | Yes |  |  |  |  |  | Yes | Yes | Yes | Yes | Yes |  |  |  |  |  |
| Stewart et al., 2022 | Yes | Yes |  |  |  |  |  |  |  |  |  |  | Yes | Yes | Yes | Yes | Yes |
| Yang, et al., 2020 | Yes | Can’t tell | Yes | Yes | Can’t tell | No | Can’t tell |  |  |  |  |  |  |  |  |  |  |
| Yuan et al., 2015 | Yes | Yes |  |  |  |  |  |  |  |  |  |  | Yes | Yes | Yes | Yes | Yes |
| Yusof 2015 | Yes | Yes | Yes | Yes | Yes | Yes | Yes |  |  |  |  |  |  |  |  |  |  |

^1^ **Qualitative studies** 1.1. Is the qualitative approach appropriate to answer the research question? 1.2. Are the qualitative data collection methods adequate to address the research question? 1.3. Are the findings adequately derived from the data? 1.4. Is the interpretation of results sufficiently substantiated by data? 1.5. Is there coherence between qualitative data sources, collection, analysis and interpretation?

^2^ **Quantitative descriptive studies** 4.1. Is the sampling strategy relevant to address the research question? 4.2. Is the sample representative of the target population? 4.3. Are the measurements appropriate? 4.4. Is the risk of nonresponse bias low? 4.5. Is the statistical analysis appropriate to answer the research question?

^3^ **Mixed methods studies** 5.1. Is there an adequate rationale for using a mixed methods design to address the research question? 5.2. Are the different components of the study effectively integrated to answer the research question? 5.3. Are the outputs of the integration of qualitative and quantitative components adequately interpreted? 5.4. Are divergences and inconsistencies between quantitative and qualitative results adequately addressed? 5.5. Do the different components of the study adhere to the quality criteria of each tradition of the methods involved?
